# AI-Based Quantitative and Objective Analysis of Aesthetic Results in Genioplasty

**DOI:** 10.1007/s00266-025-05607-z

**Published:** 2026-01-13

**Authors:** Jakob Fenske, Samuel Knoedler, Tobias Niederegger, Simon Bigus, Jan O. Voss, Rainer Pooth, Carsten Rendenbach, Max Heiland, Alexandre G. Lellouch, Leonard Knoedler

**Affiliations:** 1 Department of Oral and Maxillofacial Surgery, Charité-Universitätsmedizin Berlin, Corporate Member of Freie Universität Berlin and Humboldt-Universität zu Berlin, Berlin, Germany; 2 Division of Plastic Surgery, Department of Surgery, Yale School of Medicine, New Haven, CT USA; 3 Division of Plastic Surgery, Department of Surgery, University Hospital Regensburg, Regensburg, Germany; 4 Clinical Research and Development, ICA Aesthetic Navigation, Frankfurt am Main, Germany; 5 Division of Plastic and Reconstructive Surgery, Cedars-Sinai Medical Center, Los Angeles, CA USA; 6 Vascularized Composite Allotransplantation Laboratory, Center for Transplantation Sciences, Massachusetts General Hospital, Harvard Medical School, Boston, MA USA; 7 The Paris Cardiovascular Research Center, Team Endotheliopathy and Hemostasis Disorders, Université Paris Cité, Inserm, Paris, France; 8 Hematology Department, AP-HP, Hôpital Européen Georges Pompidou, Paris, France

**Keywords:** Genioplasty, Aesthetic surgery, Machine learning, Artificial intelligence, Aesthetic outcomes

## Abstract

**Background:**

Genioplasty and chin-augmentation are well-established procedures aimed at enhancing lower facial aesthetics. Traditionally, aesthetic outcomes have been assessed subjectively through expert opinions and patient-reported measures. The integration of artificial intelligence (AI) offers an objective approach to evaluating surgical results. This study utilizes the ICA Aesthetic Navigation AI Research Metrics Model (ICAAN® ARMM) to analyze postoperative changes in facial attractiveness, youthfulness, and skin quality following genioplasty.

**Methods:**

Pre- and postoperative full-frontal images of 50 patients undergoing osseous genioplasty were analyzed using the ICAAN® ARMM. Therefore, an array of three aesthetic scores, the Facial Aesthetic Index (FAI), Facial Youthfulness Index (FYI), and Skin Quality Index (SQI), were measured before and after surgery, with subgroup analyses by age, sex, and ethnicity. Minimally clinically important differences (MCIDs) were estimated.

**Results:**

All three aesthetic scores demonstrated improvement postoperatively, with FAI showing the greatest increase (82 (73–89) to 85 (75–92); *p *= 0.296), without showing statistical significance. Older patients (≥ 35 years) exhibited greater improvements in FAI scores compared to younger individuals (4 (1–10) vs. 1 (− 3–5); *p *= 0.028). Sex-related trends were observed, while lacking statistical significance. Ethnic subgroup analysis revealed no differences in score changes, suggesting cross-cultural applicability. Observed improvements did not exceed estimated MCIDs.

**Conclusion:**

AI-assisted aesthetic analysis offers a novel, contemporary, and objective method for assessing genioplasty outcomes. While our study suggests general aesthetic improvements following surgery, further research incorporating larger data collections and subjective patient-reported measures is necessary. AI tools hold promise as a complementary tool in aesthetic medicine, supporting both clinicians and patients in surgical decision-making.

**Level of Evidence III:**

This journal requires that authors assign a level of evidence to each article. For a full description of these Evidence-Based Medicine ratings, please refer to the Table of Contents or the online Instructions to Authors www.springer.com/00266.

**Graphical Abstract:**

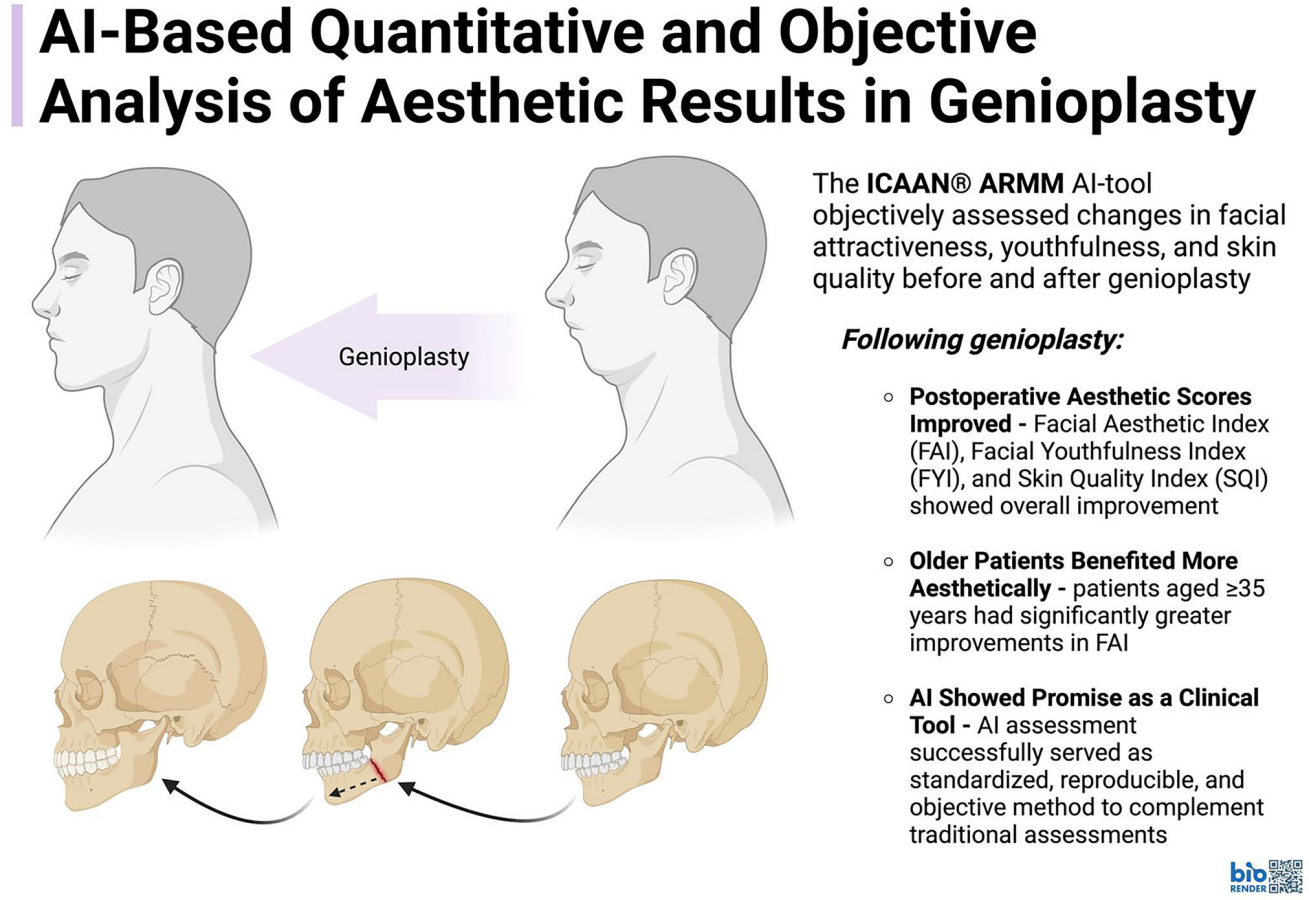

## Introduction

A well-proportioned and defined chin is a cornerstone of facial harmony, influencing both profile balance and overall aesthetic perception [1, 2]. Osseous genioplasty is an established procedure in facial plastic surgery aimed at refining the lower face’s proportions and improving overall facial balance. Whether performed to correct congenital asymmetries, age-related bone resorption, or aesthetic preferences, genioplasty can significantly impact facial harmony [3, 4]. Traditionally, subjective assessments and patient satisfaction using patient-reported outcomes measures have guided evaluations [5–8]. While this approach facilitated stakeholder engagement and assessing emotional outcomes of chin surgery [9], emerging technologies now enable objective quantification of surgical outcomes. Artificial intelligence (AI)-based tools offer a novel, standardized approach to determining postoperative changes, reducing bias and enhancing reproducibility [10, 11].

AI-assisted aesthetic analysis leverages machine learning algorithms to evaluate facial characteristics based on predefined metrics. One such system, the ICA Aesthetic Navigation AI Research Metrics Model (ICAAN® ARMM), has been developed to quantify facial attractiveness, youthfulness, and skin quality [12]. By processing detailed facial landmarks, this tool generates three key aesthetical scores, providing an objective and quantifiable measure for evaluating the outcomes of genioplasty. While AI has gained traction in facial aesthetic analysis, its application to genioplasty remains largely unexplored.

In this study, we employ the ICAAN® ARMM to assess aesthetic changes following genioplasty, using a dataset of preoperative and postoperative full-face images. By comparing facial aesthetic scores before and after surgery, we aim to objectify the extent of aesthetic improvement and identify potential variations across age and sex. This investigation contributes to the evolving role of AI in aesthetic facial surgery by offering data-driven insights into genioplasty outcomes, highlighting the potential of AI-based tools in aesthetic facial surgery.

## Materials and Methods

### Data Sourcing and Image Processing

Pre- and postoperative photographs of patients undergoing genioplasty were obtained from RealSelf.com. Since the images were publicly available, no Institutional Review Board (IRB) approval was necessary. An IRB exemption was granted by the local ethics committee at University Hospital Regensburg (24-3816-104). Available self-uploaded full-frontal face photographs were exported from the website and randomly selected and stored in March 2025. If photographs were unsuitable for analysis, e.g., due to insufficient quality, random substitution was performed. Exemplary outcomes can be accessed at https://www.realself.com/photos/genioplasty. Additionally, age range, sex, and ethnicity were collected for each patient. Subsequently, two researchers (LK and JF) independently uploaded the images into the ICAAN ® ARMM for automated aesthetic analysis, reaching identical results (Intraclass Correlation Coefficient [ICC] *ρ* = 1.0). Permission to use the ICAAN ® ARMM for this study was obtained from Caarisma® (ICA Aesthetic Navigation, Frankfurt am Main, Germany).

The ICAAN ® ARMM calculates three objective aesthetic scores: the Facial Aesthetic Index (FAI [0-100, with higher scores indicating better facial aesthetics]), the Facial Youthfulness Index (FYI [0-100, with higher scores indicating a more youthful appearance]), and the Skin Quality Index (SQI [0-100, with higher scores indicating better facial skin quality]) [13]. Briefly, the ICAAN ® ARMM uses classical machine learning algorithms to analyze 17 facial landmarks to quantify aesthetics as previously described [12, 14].

All ICAAN ® ARMM FAI, FYI, and SQI algorithms were validated on independent test sets, with established metrics such as the Pearson correlation coefficient and the coefficient of determination (*R*^2^) regarding their performance and showed strong alignment with field expert scores as indicated by the provider Caarisma® (ICA Aesthetic Navigation, Frankfurt am Main, Germany).

### Statistical Analysis

After automatic determination by the ICAAN ® ARMM, the FAI, FYI, and SQI scores of all photographs before and after surgery were stored using spreadsheet software (Microsoft Excel 2024, Microsoft Corporation, Redmond, WA, USA) and subsequently statistically analyzed using RStudio for Mac (Version 2024.09.0+375). Deviations of all three scores before and after surgery were analyzed (FAI/FYI/SQI after genioplasty)—(FAI/FYI/SQI before genioplasty), between both time points between two groups were analyzed using independent Wilcoxon–Mann–Whitney tests. The age cut-off for subgroup analysis was set at < 35 and ≥ 35 years based on the cohort’s reported age distribution. To account for potential cultural bias, ethnic subgroups (White, Latin, Asian, and Black) were compared regarding pre- to postoperative FAI/FYI/SQI changes using the Kruskal–Wallis test. A post hoc power analysis was conducted using the observed standard deviations of the pre- and postoperative differences for all scores. Cohen’s *d* was calculated and achieved power was computed. As no established minimal clinically important differences (MCIDs) exist for ICAAN® ARMM scores, MCIDs were estimated using a distribution-based approach (0.5 x baseline standard deviation of preoperative scores) [15]. All results were reported as medians and interquartile ranges (IQR). The level of statistical significance was set at a *p* value of < 0.05.

## Results

### Participant Demographics

In total, 50 patients (25 females and 25 males) were included in the study. The majority of individuals belonged to the 25–34 years age group (*n *= 24; 48%), followed by 35–44 years (*n *= 12; 24%), 18–24 years (*n *= 8; 26%), 45–54 years (*n *= 4; 8%), and 55–64 years (*n *= 2; 4%). Most patients were of White ethnicity (*n *= 35; 70%), followed by Latin (*n *= 8; 16%), Asian (*n *= 6; 12%), and Black (*n *= 1; 2%).

### Evaluation of Aesthetics Before and After Genioplasty

All three aesthetic scores improved following genioplasty. The FAI showed the highest increase from 82 (73–89) to 85 (75–92), followed by the FYI from 60 (50–72) to 62 (51–72) and the SQI from 93 (89–96) to 94 (90–96). However, the score differences were not statistically significant (Table 1). The effect sizes as indicated by Cohen’s *d* for changes in FAI (*d *= 0.21), FYI (*d *= 0.07), and SQI (*d *= − 0.03) were small, corresponding to achieved powers of 30%, 8%, and 6%, respectively. Estimated MCID were 6.8 (FAI), 7.77 (FYI), and 3.53 points (SQI). Observed median changes (FAI: +1; FYI: +1; SQI: 0) did not exceed these thresholds.

**Table 1:** Score comparison prior to and after genioplasty (IQR=interquartile range)

	Before genioplasty	After genioplasty	*p*
Facial Aesthetic Index [points], median (IQR)	82 (73–89)	85 (75–92)	0.296
Facial Youthfulness Index [points], median (IQR)	60 (50–72)	62 (51–72)	0.772
Skin Quality Index [points], median (IQR)	93 (89–96)	94 (90–96)	0.780

### Influence of Sex, Age, and Ethnicity on Genioplasty Aesthetics

In men, the FAI and FYI scores increased after genioplasty, while the SQI was slightly reduced. In women, the FAI increased after surgery, with the SQI staying constant, and the FYI reducing. No significant differences were found between both groups (Table 2).

**Table 2:** Score differences between men and women prior to and after genioplasty. (IQR=interquartile range)

	Men	Women	*p*
Facial Aesthetic Index [points], median (IQR)	1 (− 3–9)	1 (− 1–5)	0.815
Facial Youthfulness Index [points], median (IQR)	2 (− 5–15)	− 1 (− 10–5)	0.084
Skin Quality Index [points], median (IQR)	− 1 (− 3–0)	0 (− 3–3)	0.078

FAI scores significantly increased after surgery in older (≥ 35 years) patients (4 (1–10)) compared to younger (< 35 years) patients (1 (− 3–5)) (*p *= 0.028). The FYI score also increased in older patients (3 (− 9–11) vs. − 1 (− 7–6)) without reaching statistical significance. The SQI score decreased in older patients (− 1 (− 3–2)) and stayed constant in younger patients (0 (− 2–1)). Again, no statistical significance was reached (Table 3). No significant differences were found for FAI (White: 1 (− 3–7) vs. Latin: 1 (− 0.8–3.5) vs. Asian: 2 (− 0.5− 4.3) vs. Black: 17; *p *= 0.954], FYI [1 (− 5–11) vs. − 2 (− 16–6) vs. 0.5 (− 7–2) vs. 0; *p *= 0.233], and SQI [0 (− 2–2) vs. − 3 (− 3.8–0.5) vs. − 0.5 (− 1.5–1.5) vs. 1; *p *= 0.286) when comparing their deviations across different ethnic groups.

**Table 3:** Score differences of younger (< 35 years) and older (≥ 35years) patients prior to and after genioplasty. (IQR=interquartile range)

	Younger (<35 years)	Older (≥35 years)	*p*
Facial Aesthetic Index [points], median (IQR)	1 (− 3–5)	4 (1–10)	**0.028**
Facial Youthfulness Index [points], median (IQR)	− 1 (− 7–6)	3 (− 9–11)	0.537
Skin Quality Index [points], median (IQR)	0 (− 2–1)	− 1 (− 3–2)	0.495

*p* < .05 is given in bold

## Discussion

The present study utilized an AI-based analysis tool to objectively assess outcomes following genioplasty. By leveraging automated facial analysis, we aimed to quantify postoperative changes in facial aesthetics, youthfulness, and skin quality. Our findings indicate a general trend toward improved facial aesthetics post-genioplasty, with nuanced differences observed across age and sex groups. As the first AI-driven study performed on aesthetic genioplasty outcomes, these preliminary insights underscore the potential of AI-driven evaluations in aesthetic surgery.

### Overall Enhanced Facial Aesthetics After Genioplasty

The presented results demonstrate an overall increase in facial aesthetic scores after genioplasty. The FAI exhibited the most notable improvement, followed by the FYI and SQI. These findings align with the primary goals of genioplasty: enhancing lower facial balance to achieve a more aesthetic and harmonious profile [16]. While statistical significance was not reached, the upward trend across all three scores suggests that the procedure indeed positively impacts facial appearance. Nevertheless, these changes were small in magnitude without exceeding estimated MCID threshold and were limited in power to reliably detect small differences. One possible explanation for the lack of statistical significance is the inherent variability in patient anatomy and aesthetic preference communication prior to surgery, although no data for analyzing this assumption are available. Genioplasty outcomes depend on multiple factors, including initial facial structure, surgical technique, and postoperative healing [17–19]. Moreover, locoregional incremental improvements may not always translate into overall statistically significant changes. Interestingly, a previous study reported that subjective aesthetic satisfaction was increased, if genioplasty was performed with orthognathic surgery, rather than alone, which may contribute to aesthetic scores only being slightly elevated after isolated genioplasty [18]. Future research with larger sample sizes and additional covariates may help delineate the specific contributions of surgical modifications to facial aesthetics.

### Gender and Age-Specific Differences in Aesthetic Perception

Our study highlights notable sex- and age-related variations in aesthetic perception post-genioplasty [20–22]. Men generally benefited from an increase in perceived facial aesthetics and youthfulness, whereas women exhibited a more variable response. These differences likely stem from the distinct cultural and biological determinants of facial attractiveness. A strong, well-defined jawline is widely associated with masculinity, strength, and dominance, making chin augmentation particularly beneficial for male patients [23, 24]. In fact, chin and jawline proportions have been deemed one of the key drivers in male facial aesthetics [25]. This finding aligns with previous research indicating that a prominent chin enhances male attractiveness, reinforcing traditional aesthetic ideals [26–28]. Age also played a crucial role in shaping postoperative aesthetic outcomes. Older patients (≥ 35 years) experienced greater improvements in facial aesthetic scores compared to their younger counterparts. This effect may be attributable to the age-related resorption of the mandible, which can lead to an overall loss of lower facial definition [29]. Genioplasty may counteract these changes, restoring a youthful and well-proportioned facial contour. Furthermore, societal perceptions of aging in men may explain why older men with prominent chins are often regarded as more attractive, with prior studies suggesting that male attractiveness peaks in midlife, matching with our findings of increased facial aesthetic scores in this cohort [30]. Besides that, we examined differences regarding cultural bias by comparing score deviations across different included ethnicities. Although ethnic group allocation varied due to availability and the analysis therefore needs to be interpreted exploratorily, no significant differences were found across ethnic groups. Therefore, the initial results imply that the employed algorithm may be useful to assess and quantify different cultural beauty standards.

### Integration of AI in Facial Aesthetic Surgery and Plastic Surgery

The integration of AI tools such as the ICAAN® ARMM represents a paradigm shift in aesthetic medicine [31]. Our findings demonstrate the feasibility of AI-driven evaluations, offering an objective and standardized approach to assessing surgical outcomes. This technology holds promise for both clinicians and patients by providing quick, reproducible, and bias-free aesthetic assessments. In clinical practice, AI tools could complement traditional assessment methods, aiding surgeons in preoperative planning and postoperative evaluations [32]. By offering quantitative feedback, AI may help refine surgical techniques and optimize patient outcomes. Additionally, AI-based aesthetic analysis can serve as a valuable adjunct to patient-reported outcome measures, ensuring a holistic evaluation of surgical success. Despite these advantages, AI-driven assessments should not replace expert judgment or patient preferences. Aesthetic ideals vary across cultures, personal tastes, and societal norms, necessitating a personalized approach to surgical planning [33]. To enhance AI’s applicability in genioplasty, future iterations should incorporate larger datasets, diverse ethnic groups, and additional facial parameters. Expanding the AI model’s scope will enable a more comprehensive understanding of what constitutes an aesthetically favorable chin-augmentation outcome. In fact, contemporary plastic surgery increasingly relies on digital technologies for objective pre- and postoperative measurements across disciplines, such as aesthetic, reconstructive, hand, and wound surgery, laying the groundwork for future expansions of this study toward additional objective clinical metrics (e.g., 3D imaging and wound-area quantification) [12, 14, 34]. Furthermore, the integration of validated PROMs, such as SCAR-Q, BREAST-Q, and BODY-Q, enables systematic evaluation of patient-centered outcomes and could navigate future research efforts [35–37].

### Limitations

While our study provides valuable insights, several limitations must be acknowledged. First, the retrospective nature of our dataset introduces potential biases in patient selection and image quality. Since these images were self-submitted, variations in lighting, camera angles, and facial expressions may have influenced the AI-generated scores. Consequently, results need to be interpreted exploratorily and hypothesis-generating, aiding in design of future prospective and standardized investigations. Moreover, reliance on these photographs may introduce selection bias, as these patients may not be fully representative for the general genioplasty population, thereby limiting generalizability. Second, the relatively small sample size limits the generalizability of our findings. Post hoc power analysis indicated that the sample size was underpowered to detect small changes, with achieved powers between 6 and 30%. The magnitude of change did not exceed estimated MCIDs. These findings highlight the need for prospectively powered, larger studies to validate MCID thresholds for this model. Larger cohorts with a more balanced representation of age, sex, ethnic diversity, and surgical, as well as personal covariates are needed to confirm and expand upon our results. Additionally, the selective lack of statistical significance highlights the need for increased sample power in future studies. Third, no matched non-surgical controls were included due to reliance on a retrospective framework with publicly available images. This reinforces the exploratory character of the study, which were further contextualized by MCID thresholds. Fourth, while AI-based aesthetic analysis offers objective quantification, it does not account for individual patient satisfaction or psychosocial factors influencing perceived attractiveness. In addition, there were no data available in the use of alloplastic implants which have shown inferior outcomes compared to osseous genioplasties [38]. Future research should integrate AI evaluations with patient-reported outcomes and expert assessments to provide a more holistic measure of surgical success.

## Conclusion

This exploratory study demonstrates the feasibility of applying AI-assisted aesthetic analysis to evaluate outcomes after genioplasty. Small postoperative improvements in aesthetic scores were observed, particularly among older patients, although changes lacked statistical significance and remained below estimated MCID thresholds, underscoring the need for larger, prospective studies to establish concurrent MCID thresholds. Nonetheless, the study highlights the potential usefulness of AI tools for objective and reproducible aesthetic assessment in facial plastic surgery. Future research using larger, prospectively collected datasets with standardized imaging is needed to validate these preliminary observations and to more precisely determine the role of AI-based analyses in evaluating genioplasty outcomes.
